# In Vitro Toxicity of Cetalkonium Chloride on Corneal Epithelial Cells

**DOI:** 10.3390/pharmaceutics17040522

**Published:** 2025-04-16

**Authors:** Joo-Hee Park, Choul Yong Park

**Affiliations:** 1 Department of Ophthalmology, Dongguk University Ilsan Hospital, Goyang 10326, Republic of Korea; pjoohee9@gmail.com; 2 Department of Ophthalmology, Samsung Medical Center, Sungkyunkwan University School of Medicine, Suwon 06351, Republic of Korea

**Keywords:** cetalkonium chloride, cornea, epithelium, toxicity, preservative

## Abstract

**Objective**: To investigate the toxicity of cetalkonium chloride (CKC) on primary cultured human corneal epithelial cells (HCECs). **Methods**: HCECs were subjected to various concentrations (0.03125 × 10^−4^ to 2.0 × 10^−4^% (*w/v*)) of CKC for durations ranging from 24 to 72 h. Cell viability was evaluated using the CCK-8 kit along with live and dead cell staining. Intracellular reactive oxygen species (ROS) levels were measured 20 min following CKC exposure. Observations of changes in cell morphology, cytoplasmic actin filaments, and mitochondrial distribution were conducted using immunocytochemistry and MitoTracker assays. Protein expression levels related to cell survival pathways, including mTOR, ERK, Akt, Bcl-xL, and BAX, were examined via Western blot analysis. **Results**: CKC exhibited dose-dependent toxicity in HCECs. Exposure to CKC concentrations below 0.125 × 10^−4^% resulted in no significant decrease in HCEC viability for up to 72 h. Conversely, exposure to CKC at concentrations of 1.0 × 10^−4^% or higher led to significantly decreased HCEC viability. Following exposure to higher concentrations of CKC, elevated levels of intracellular ROS and LDH release were observed. This toxicity was further characterized by decreased levels of phosphorylated mTOR, phosphorylated Akt, phosphorylated ERK, and Bcl-xL, as well as an increase in BAX expression. As the CKC concentration increased, HCECs decreased in size, and mitochondria displayed a loss of characteristic punctate staining. **Conclusions**: Our findings indicated that exposure to CKC caused significant toxicity in HCECs, which varied with concentration and duration of exposure. This toxicity was associated with an increase in ROS, mitochondrial alterations, and a decline in activity of the cell survival pathways.

## 1. Introduction

Quaternary ammonium compounds, such as benzalkonium chloride (BAK), are widely adopted as preservatives in eye drops due to their broad-spectrum antimicrobial properties. They effectively combat a diverse array of microorganisms, including both Gram-positive and Gram-negative bacteria, and fungi [1,2]. These compounds achieve this by disrupting the microbial cells’ lipid membranes, leading to cell death [2]. Furthermore, quaternary ammonium compounds exhibit high water solubility, facilitating their inclusion in aqueous formulations [1,2]. They also enhance the penetration of active pharmaceutical ingredients into the cornea by disrupting the hydrophobic barrier of the corneal epithelium [3]. The synthesis of antimicrobial efficacy, solubility, and the ability to improve drug delivery renders quaternary ammonium compounds an optimal choice for preserving ophthalmic solutions [1,3,4].

In the field of ophthalmology, BAK is the most prevalent quaternary ammonium compound utilized in eye drops [1]. Nevertheless, due to emerging concerns regarding its ocular toxicity, ongoing research aims to identify alternative compounds. Cetalkonium chloride (CKC) has been proposed as a potential substitute [1,4,5,6]. In comparison to BAK, CKC is distinguished by an alkyl group with a chain length of C16 and may be present in trace amounts in the excipient mixture containing BAK [7]. Previously, it was reported that CKC accumulates in the lipid layer of the tear film model, stabilizing it in a concentration-dependent manner, while BAK’s interaction with the lipid layer compromises the stability of the tear film model [7].

CKC has been incorporated into cationic emulsion eye drops, such as those containing latanoprost or cyclosporine nano-emulsion [8]. It imparts a positive charge, which enhances the bio-adhesive properties of these cationic nano-emulsions on the negatively charged ocular surface. Such enhancement facilitates the penetration of active ingredients in cationic nano-emulsions into ocular tissues [8].

In this study, we investigated the potential toxicity of CKC in cultured human corneal epithelial cells (HCECs). We observed changes in the viability of HCECs, the generation of intracellular reactive oxygen species, mitochondrial alterations, and modifications in cellular pathways related to survival, depending on the dosage and exposure duration to CKC.

## 2. Materials and Methods

### 2.1. Human Corneal Epithelial Cell (HCEC) Culture

The primary culture of HCECs (Cat. PCS-700-010) was obtained from ATCC (American Type Culture Collection, Manassas, VA, USA). These cells were resuspended in corneal epithelial cell basal medium (serum- and calcium-free) and supplemented with a growth kit provided by ATCC. They were plated in 75 cm^2^ tissue flasks with an FNC coating mix (Athena Enzyme Systems, catalog number 0407, Baltimore, MD, USA) and maintained at 37 °C in a 5% CO_2_ and 95% air humidified atmosphere. The culture medium was replaced every 3 days, and the cells were subcultured using 0.05% Trypsin-EDTA (GibcoBRL, Grand Island, NY, USA). Cells with a passage number of ≤5 were utilized in this study.

### 2.2. Preparation of Cetalkonium Chloride (CKC)

Cetalkonium chloride (CKC; >97% in dried material), obtained from Merck (Catalog No. B4136) KGaA, Darmstadt, Germany, was dissolved in methanol (Merck) for serial dilution, creating a vehicle concentration of 0.004% (*v/v*) methanol. A 10% (*w/v*) stock solution and freshly prepared working solutions of CKC were utilized on the experiment day.

### 2.3. Cell Viability Assay

Cell viability of HCECs was assessed using the Cell Counting Kit-8 (CCK-8; Dojindo Laboratories, Kumamoto, Japan). HCECs were seeded in a 96-well plate at a density of 1 × 10^4^ cells/well. After cell adherence, CKC was applied at concentrations of 0, 0.03125, 0.0625, 0. 125, 0.25, 0.5, 1.0, 2.0, and 4.0 × 10^−4^% (*w/v*) for durations ranging from 24 to 72 h. After incubation, 10 μL of CCK-8 reagent was added to each well. Following 4 h of incubation with the reagent at 37 °C, the absorbance at 450 nm was measured using a microplate reader.

### 2.4. Live and Dead Cell Staining

To obtain both quantitative and qualitative data, the viability and cytotoxicity of CKC-treated HCECs were evaluated using a live/dead viability/cytotoxicity kit (Molecular Probes, Cat. L3224, Thermo Fisher Scientific, Rochester, NY, USA). HCECs were cultured in confocal dishes and treated with CKC at concentrations of 0.25, 0.5, 1.0, and 2.0 × 10^−4^% (*w/v*) for 24 h. Prior to staining, the cells were washed twice with DPBS, and the staining reagents, calcein AM (final concentration: 2 μM) and EthD-1 (final concentration: 4 μM), were diluted in DPBS, as per the manufacturer’s protocol. The HCECs were then incubated with this staining solution at 37 °C for 30 min in the dark to prevent photobleaching. Post-incubation, the cells were gently washed with DPBS to remove excess stain, and fluorescence imaging was conducted using a confocal live imaging system (Leica Microsystems CMS GmbH, Mannheim, Germany).

### 2.5. Measurement of Reactive Oxygen Species

To measure intracellular reactive oxygen species (ROS), we employed the DCFDA/H2DCFDA-Cellular ROS Assay Kit by Abcam (Cat. ab113851; Cambridge, UK). HCECs were exposed for 20 min to varying concentrations of CKC (0, 0.0156, 0.0313, 0.0625, 0.125, 0.25, 0.5, 1.0, and 2.0 × 10^−4^% *w/v*). After treatment, the media were discarded, and the cells were washed with 100 µL/well of 1× ROS buffer according to the manufacturer’s protocol. In a dark environment at 37 °C, cells were stained for 45 min by adding 100 µL/well of a 20 µM solution of 2′,7′-dichlorofluorescin diacetate (DCFDA). Afterward, the cells were removed from the stain and rinsed with 100 µL/well of 1× ROS buffer. Fluorescence was immediately quantified at 485 nm excitation/535 nm emission using an endpoint assay.

### 2.6. Lactate Dehydrogenase Assay

The cellular toxicity of each experimental condition was measured by a colorimetric assay using a lactate dehydrogenase (LDH) cytotoxicity detection kit (Takara Bio Inc., Shiga, Japan), and experiments were performed according to the manufacturer’s protocol. The LDH assay is a quantitative analysis of LDH secreted from dead cells. HCECs were cultured and incubated at 1 × 10^4^ cells/well in a 96-well plate. After cell attachment, cells were exposed to 0.03125, 0.0625, 0.125, 0.25, 0.5, 1.0, 2.0, and 4.0 × 10^−4^% *w/v* of CKC for 24, 48, and 72 h. After the appropriate incubation period, the activity of LDH released by the cells was measured in supernatants collected after each incubation time, adding 10% (*v/v*) of LDH solution. The wells to which CKC was not added and those to which 1% Triton X-100 was added were used as the negative and positive controls, respectively, and absorbance was measured at 490 nm.

### 2.7. MitoTracker Assay

To evaluate mitochondrial activity and localization in live cells, MitoTracker Deep Red FM from Molecular Probes (Cat. M22426), a mitochondria-selective probe, was used, which allowed for subsequent immunostaining. HCECs were cultured at a density of 4 × 10^4^ cells/mL in 4-well Nunc Lab-Tek II chamber slides (Thermo Fisher Scientific) and treated with CKC for 24 h. The cells were then incubated with MitoTracker solution (final concentration of 100 nM) at 37 °C for 30 min in darkness to prevent photobleaching. Post-incubation, the cells were washed gently with DPBS to remove excess dye and fixed with 10% formaldehyde at RT for 10 min. Cell permeabilization was conducted using 0.1% Triton X-100 for 5 min at RT. After rinsing with DPBS, nonspecific binding sites were blocked using 1% bovine serum albumin in DPBS at RT for 30 min. The chamber slides were then incubated overnight at 4 °C with Goat anti-Zo1 (1:200; Abcam; Cat. ab190085), followed by washing with DPBS and incubation with Alexa488-conjugated donkey anti-goat antibody (1:1000; Abcam; Cat. ab150129) at RT for 1 h. After multiple washing steps, tetramethylrhodamine isothiocyanate (TRITC)-conjugated phalloidin (1 µg/mL; Sigma-Aldrich) was used to stain F-actin. Cell nuclei were counterstained with 4ʹ,6-diamidino-2ʹ phenylindole (DAPI, Roche; Cat. 10236276001; Mannheim, Germany). Fluorescence images were captured with a confocal microscope (Leica Microsystems CMS GmbH).

### 2.8. Western Blot Assay for Cell Survival Signals

All CKC-treated cells were lysed using ice-cold radioimmunoprecipitation assay buffer (50 mM TrisHCl (pH 8.0), 150 mM NaCl, 1% NP-40, 0.5% deoxycholate, and 0.1% sodium dodecyl sulfate) for 30 min. The cellular debris was removed by centrifugation at 16,000× *g* for 10 min. Equal amounts (20 μg) of total cell protein were separated by sodium dodecyl sulfate polyacrylamide gel electrophoresis (SDS-PAGE) and then transferred onto a polyvinylidene difluoride (PVDF) membrane (Millipore Corporation, Billerica, MA, USA). The membrane was blocked with 3% BSA in TTBS buffer (10 mM Tris, pH 8.0, 150 mM NaCl, and 0.1% Tween 20) for 1 h at RT. Subsequently, the membranes were incubated overnight at 4 °C with the following primary antibodies: rabbit anti-mammalian target of rapamycin (mTOR; 1:1000; Cell Signaling; Cat. 5536; Danvers, MA, USA), rabbit anti-phospho-mTOR (1:1000; Cat. 2983; Cell Signaling), rabbit anti-Akt (1:1000; Cell Signaling; Cat. 9272), rabbit anti-phospho-Akt (1:1000; Cell Signaling; Cat. 4060), rabbit anti-P-p44/42 MAPK (ERK 1/2; 1:1000; Cell Signaling; Cat. 4370), rabbit anti-p44/42 MAPK (ERK 1/2; 1:1000; Cell Signaling; Cat. 4695), rabbit anti-Bcl-2-associated X protein (BAX; 1:1000; Cell Signaling; Cat. 2772), rabbit anti-B-cell lymphoma (Bcl)-/xL (1:1000; Cell Signaling; Cat. 2764), and mouse anti-β-actin (1:50,000; Sigma-Aldrich; Cat. A5441). The membranes were then incubated with horseradish peroxidase-conjugated secondary antibodies at RT for 1 h. Blots were developed with Pierce enhanced chemiluminescence (ECL) substrate (Thermo Fisher Scientific; Cat. 32106) and visualized with a Fusion Pulse 6 chemiluminescence system (Vilber Lourmat, Marne-la-Vallee, France). Densitometric analysis was performed using Multi Gauge V3.0 software (Fujifilm Life Science, Tokyo, Japan). All experiments were conducted at least in triplicate.

### 2.9. Statistical Analysis

The data were presented as the mean ± standard error. Statistical significance was determined using analysis of variance (ANOVA) followed by Dunnett’s multiple comparison test. *p*-values less than 0.05 were considered statistically significant. Analyses were performed using GraphPad Prism Ver. 9.3.1 (GraphPad Software Inc., La Jolla, CA, USA).

## 3. Results

### 3.1. Cell Viability After Exposure to CKC

**Figure 1:**
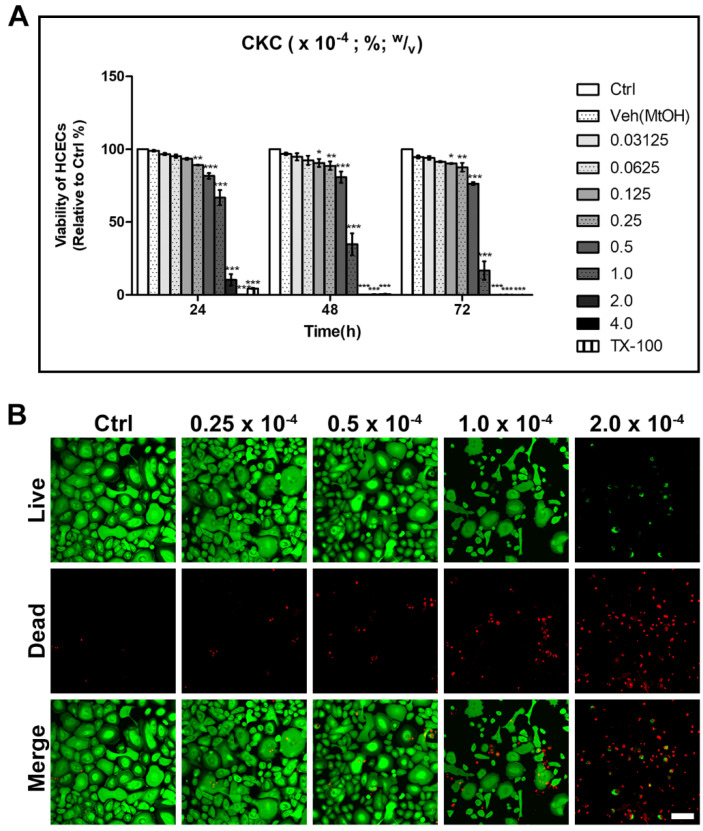
(**A**) The results from the Cell Counting Kit-8 (CCK8) assay for corneal epithelial cell cultures exposed to cetalkonium chloride (CKC) at concentrations ranging from 0.03125 × 10^−4^% to 4.0 × 10^−4^% over 24, 48, and 72 h revealed both dose- and time-dependent toxicity associated with CKC. Exposure to CKC concentrations below 0.125 × 10^−4^% did not significantly reduce cell viability up to 72 h. In contrast, CKC concentrations equal to or greater than 0.125 × 10^−4^% exhibited significant toxicity after 48 h. At a CKC concentration of 1.0 × 10^−4^%, cell viability decreased by more than 50%, and at concentrations of 2.0 × 10^−4^% or higher, almost no viable cells remained after 48 h. (**B**) In addition, live/dead cell staining revealed dose-dependent changes in live and dead cell populations after 24 h of exposure to various CKC concentrations. Values are presented as mean ± SEM and were obtained from three independent experiments, with each being performed in triplicate. Scale bar: 100 µm. * *p* < 0.05, ** *p* < 0.01, and *** *p* < 0.001.

When HCECs were exposed to CKC, cytotoxicity was proportional to both the exposure concentration and duration. The corneal epithelium, keratocytes, and corneal endothelium all exhibited similar toxicity trends. Specifically, exposure to CKC concentrations below 0.125 × 10^−4^% showed no significant decrease in cell viability up to 72 h. At CKC concentrations up to 0.5 × 10^−4^%, approximately 70% of cells survived even after 72 h of exposure. However, at concentrations starting from 1.0 × 10^−4^%, over 50% of cells died after 48 h. Exposure of HCECs to CKC at a concentration of 2.0 × 10^−4^% resulted in 90% cell death after 24 h and almost complete cellular death after 48 h (Figure 1).

### 3.2. LDH and ROS Assay

**Figure 2:**
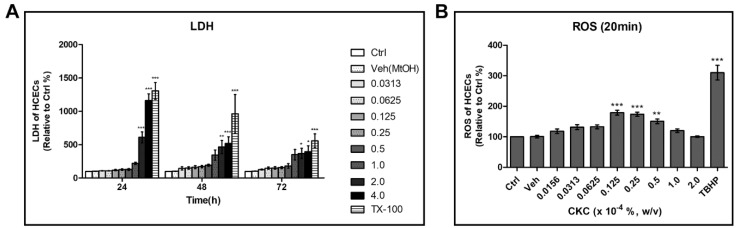
(**A**) Lactate dehydrogenase (LDH) assay indicated significant death of HCECs following exposure to high concentrations of cetalkonium chloride (CKC). (**B**) Intracellular reactive oxygen species (ROS) levels exhibited significant changes after exposure to CKC concentrations ranging from 0.015625 × 10^−4^% to 2.0 × 10^4^% for 20 min in HCECs. Ctrl: negative control cultured in media only. Triton X (TX-100) and TBHP were used as positive controls. Values are presented as mean ± SEM and were obtained from three independent experiments, and each experiment was performed in triplicate. * *p* < 0.05, ** *p* < 0.01, and *** *p* < 0.001.

Following exposure to CKC, LDH release from HCECs increased proportionally with the CKC concentration. There was a sharp increase in LDH release detected at an exposure to 1.0 × 10^−4^%. The most pronounced increase in LDH occurred after 24 h of exposure, and the degree of LDH increase declined after 48 and 72 h, which is presumed to be the result of LDH denaturation over time. ROS was measured at 20 min after CKC exposure, the earliest time point possible considering the experimental technique. Significant increases in ROS were observed after exposure to CKC concentrations from 0.125 × 10^−4^% to 0.5 × 10^−4^%; however, at higher concentrations, the cellular destruction was so extensive that significant intracellular ROS increases could not be assessed (Figure 2).

### 3.3. MitoTracker Assay

**Figure 3:**
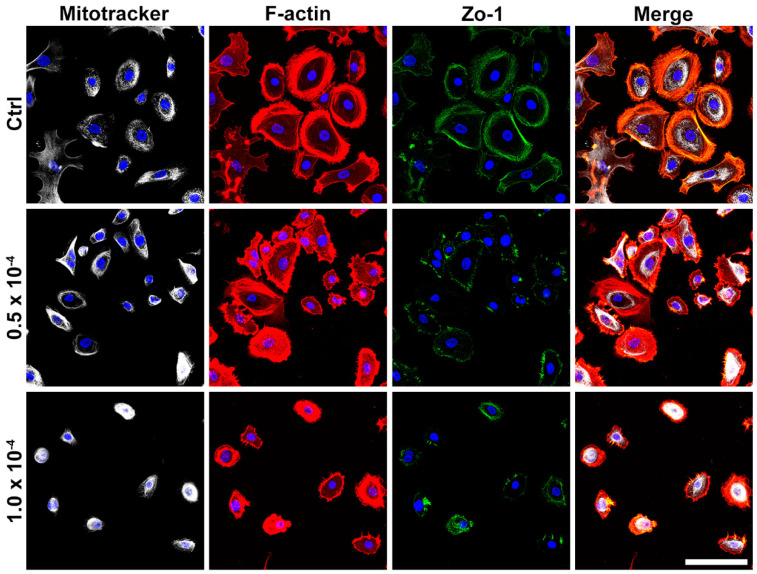
MitoTracker assay of HCECs after exposure to CKC. In normal HCECs, mitochondria were uniformly distributed in a granular pattern around the nucleus. When exposed to CKC, the mitochondria in HCECs became more densely concentrated around the nucleus and lost their granular pattern. Upon exposure to higher concentrations of CKC, HCECs exhibited severe shrinkage in cell size, with mitochondria intensely concentrated very close to the nucleus. F-actin stained the actin cytoskeleton and Zo-1 stained tight junctions. Scale bar = 100 µm.

MitoTracker Deep Red FM is a fluorescent dye utilized to visualize mitochondria, with a brighter signal indicating a higher mitochondrial concentration in the observed area. In the control group, mitochondria exhibited bright and punctate staining in the perinuclear cytoplasm. However, with increasing concentrations of CKC exposure, a noticeable reduction in both cell number and size occurred, and mitochondria lost their punctate staining characteristics, appearing diffuse. This diffuse staining pattern is indicative of mitochondrial damage or a significant loss of membrane potential (Figure 3).

### 3.4. Effect of CKC on HCEC Survival Pathway

**Figure 4:**
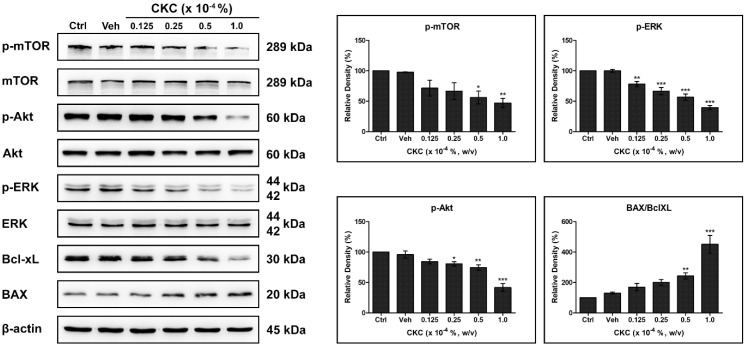
Effect of CKC on the cell survival pathway of HCECs. The expression levels of phosphorylated mTOR, phosphorylated Akt, and Bcl-xL demonstrated a dose-dependent decrease in HCECs after exposure to CKC for 24 h, accompanied by increased expression of BAX. Values are presented as mean ± SEM and were obtained from three independent experiments, and each experiment was performed in triplicate. * *p* < 0.05, ** *p* < 0.01 and *** *p* < 0.001.

Exposure to CKC led to the inhibition of mTOR, ERK, and Akt phosphorylation, which are known to facilitate cell division and migration in HCECs. This inhibitory effect was more pronounced at higher CKC concentrations. Moreover, the expression of Bcl-xL, which suppresses cell apoptosis, was downregulated, while the expression of BAX, which promotes apoptosis, was upregulated in CKC-exposed HCECs. Thus, the BAX/Bcl-xL ratio increased in a dose-dependent manner following CKC exposure (Figure 4).

## 4. Discussion

In this study, we observed that CKC induced toxicity in HCECs in a dose- and time-dependent manner. Notably, exposure to CKC concentrations of 1.0 × 10^−4^% or higher led to substantial toxicity, and at concentrations of 2.0 × 10^−4^%, virtually all HCECs perished.

CKC shares several properties with BAK, attributed to the presence of active quaternary ammonium components in both. The threshold concentration of BAK at which toxicity is noted is approximately 0.005%; conversely, BAK is frequently utilized in topical ophthalmic formulations at concentrations ranging from 0.04% to 0.02% [1]. In both tissue culture and animal models, BAK has been demonstrated to diminish the survival of cells in the corneal, conjunctival, trabecular meshwork, and ciliary epithelium [9,10,11,12]. Furthermore, BAK induces lymphocyte infiltration into the conjunctival epithelium and stroma in animal models and elevates levels of inflammatory markers in ocular tissues [11,12]. Additionally, BAK leads to mitochondrial dysfunction [5]. It also inhibits mitochondrial ATP synthesis in a concentration-dependent manner within a low micromolar range (IC_50_, 0.0002%) and blocks mitochondrial respiration with an IC_50_ of 0.0004% within 10 min of administration. The variability in toxicity thresholds of BAK observed in previous studies, ranging from 0.0004% to 0.005%, can be attributed to differing experimental conditions. Generally, in cell-based experiments, toxicity becomes apparent even at lower concentrations because, unlike in vivo, there is no dilution effect from tears, allowing the drug to maintain a consistent concentration and impact the cells over an extended period.

Due to the absence of prior studies on the toxicity mechanism of CKC in the field of ophthalmology, we hypothesized that CKC might exert toxicity on corneal cells through a mechanism similar to that of the previously reported BAK. As BAK has been demonstrated to elevate ROS levels and induce mitochondrial damage, this study found that high concentrations of CKC resulted in increased ROS and diminished mitochondrial function in the corneal epithelium, keratocytes, and endothelium.

CKC is primarily utilized in eyedrops to create cationic emulsions. Cationic emulsions provide several benefits for ocular drug delivery, such as improved retention due to the positive charge interacting with the negatively charged ocular surface, leading to better adhesion and a prolonged retention time [8,13]. This electrostatic attraction also enhances drug penetration by ensuring even distribution across the ocular surface, thus increasing the drug’s absorption into corneal and conjunctival tissues [8,13]. Consequently, the need for frequent drug administration can be reduced, simplifying the treatment regimen for patients. These characteristics make cationic emulsions an effective vehicle for delivering therapeutic agents in the treatment of various ocular conditions, including dry eye disease and keratitis. Cationic emulsion-based eye drops are available in the ophthalmic market and have been positively reviewed. Cationorm^®^ (Santen Ltd., Osaka, Japan), a clinically well-established lipid treatment for evaporative dry eye, is one example [14,15,16]. It is a preservative-free cationic nano-emulsion that contains mineral oils, cetalkonium chloride, tyloxapol, poloxamer 188, tromethamine, and glycerin. The exact concentration of CKC in Cationorm has not been disclosed. Additionally, another cationic emulsion formulation that contains 0.1% (1 mg/mL) cyclosporin A (Ikervis; Santen SAS, Evry, France) was approved by the European Medicines Agency for the treatment of severe keratitis in adults with dry eye disease [17,18]. Ikervis contains 0.05 mg of CKC in 1 mL of emulsion, which means 0.005% solution. Thanks to the electrostatic attraction between the cationic emulsion and the negative charge on ocular surface cells, spreading upon instillation is optimized. Both Cationorm^®^ and Ikervis have demonstrated efficacy in treating mild to moderate dry eye disease.

However, due to the potential concentration-dependent toxicity of CKC confirmed in this study, special attention is necessary when formulating cationic emulsions containing CKC. Our findings suggest that eye drops containing CKC concentrations of 1.0 × 10^−4^% (0.0001%) or higher have the potential to cause significant damage to the corneal epithelium. In this study, significant increases in ROS and notable mitochondrial changes in HCECs were observed after exposure to CKC concentrations from 0.125 × 10^−4^% to 0.5 × 10^−4^%. This phenomenon is akin to findings observed in cells exposed to BAK, which was noted earlier for its inhibition of mitochondrial function within a low concentration range (0.0002–0.0004%). Our findings may help explain why a previous study observed significant increases in LDH levels and a reduction in mitochondrial function when a human corneal epithelial cell line was exposed to a 10-fold diluted Cationorm for 5, 15, and 30 min [19].

Consequently, the concentration of CKC included in the eye drop should be restricted to less than 0.125 × 10^−4^% to ensure safety for the corneal epithelial layer. This concentration is approximately 10 times more diluted than that of BAK.

Our study presents several limitations. Methanol was used as a vehicle to dissolve CKC, and while the toxicity study accounted for the effects of methanol as a vehicle, its potential to enhance CKC’s toxic effects cannot be ruled out. The toxic effects observed in cultured corneal epithelial cells in this study may warrant further validation through animal experiments. Additionally, cultured primary corneal cells may exhibit higher sensitivity to toxic effects compared to corneal tissue. In vivo, the presence of tears, their various components, and negatively charged mucin, whether dissolved or anchored, could alter the toxicity of CKC on corneal epithelial cells. As this study primarily focused on short-term toxicity using cultured cells, further in vivo research is essential to evaluate the effects of prolonged CKC exposure over weeks or months.

## 5. Conclusions

In conclusion, this experiment elucidated the potential concentration-dependent toxicity of CKC, which is being explored as a substitute for BAK and is already incorporated in clinical eye drop formulations, using HCECs. Similar to BAK, CKC can induce mitochondrial damage, ROS production, and cellular destruction in HCECs.

## Data Availability

The datasets generated and/or analyzed during the current study are available from the corresponding author upon reasonable request.
